# Impact of the ABCDE triage in primary care emergency department on the number of patient visits to different parts of the health care system in Espoo City

**DOI:** 10.1186/1471-227X-12-2

**Published:** 2012-01-04

**Authors:** Jarmo Kantonen, Ricardo Menezes, Tuula Heinänen, Juho Mattila, Kari J Mattila, Timo Kauppila

**Affiliations:** 1 City of Vantaa, (Peltolantie 2 D), Vantaa, (01300), Finland, and Attendo-Medone LTD, (Porkalankatu 22), Helsinki, (00181), Finland; 2 Emergency services project, City of Espoo, (Siltakatu 11), Espoo, Finland, (02070), and Medivida LTD, (Laivakatu 3), Helsinki, (00150), Finland; 3 Emergency services project, Director of Health Services, City of Espoo, City of Espoo, (Siltakatu 11), Espoo, (02070), Finland; 4 Helsinki University Central Hospital, HUS, (Haartmaninkatu 4), Helsinki, (00029), Finland; 5 Medical School, University of Tampere, (Lääkärinkatu 1), Tampere, (33014), Finland, and Center of General Practice, Hospital District of Pirkanmaa, (Teiskontie 35), Tampere, (33521), Finland; 6 City of Vantaa, (Peltolantie 2 D), Vantaa, (01300), Finland, Network of Academic Health Centres, Departments of General Practice and Primary Healthcare, HUS Institute of Clinical Medicine, (Tukholmankatu 8B), Helsinki, (00014), Finland, and Department of National Public Health, Hjelt-institute, University of Helsinki, (Mannerheimintie 172) Helsinki, (00014), Finland

## Abstract

**Background:**

Many Finnish emergency departments (ED) serve both primary and secondary health care patients and are therefore referred to as combined emergency departments. Primary care doctors are responsible for the initial assessment and treatment. They, thereby, also regulate referral and access to secondary care. Primary health care EDs are easy for the public to access, leading to non-acute patient visits to the emergency department. This has caused increased queues and unnecessary difficulties in providing immediate treatment for urgent patients. The primary aim of this study was to assess whether the flow of patients was changed by implementing the ABCDE-triage system in the EDs of Espoo City, Finland.

**Methods:**

The numbers of monthly visits to doctors were recorded before and after intervention in Espoo primary care EDs. To study if the implementation of the triage system redirects patients to other health services, the numbers of monthly visits to doctors were also scored in the private health care, the public sector health services of Espoo primary care during office hours and local secondary health care ED (Jorvi hospital). A face-to-face triage system was applied in the primary care EDs as an attempt to provide immediate treatment for the most acute patients. It is based on the letters A (patient sent directly to secondary care), B (to be examined within 10 min), C (to be examined within 1 h), D (to be examined within 2 h) and E (no need for immediate treatment) for assessing the urgency of patients' treatment needs. The first step was an initial patient assessment by a health care professional (triage nurse). The introduction of this triage system was combined with information to the public on the "correct" use of emergency services.

**Results:**

After implementation of the ABCDE-triage system the number of patient visits to a primary care doctor decreased by up to 24% (962 visits/month) as compared to the three previous years in the EDs. The Number of visits to public sector GPs during office hours did not alter. Implementation of ABCDE-triage combined with public guidance was associated with decreased total number of doctor visits in public health care. During same period, the number of patient visits in the private health care increased. Simultaneously, the number of doctor visits in secondary health care ED did not alter.

**Conclusions:**

The present ABCDE-triage system combined with public guidance may reduce patient visits to primary health care EDs but not to the secondary health care EDs. Limiting the access of less urgent patients to ED may redirect the demands of patients to private sector rather than office hours GP services.

## Background

Non acute and non-urgent visits to the emergency department (ED) may cause significant problems since they consume resources that should be allocated for acute patients [1-4]. Triage has, in part, been developed in order to allocate resources [3,4]. Emergency departments around the world use different triage systems to assess the severity of incoming patients' conditions and assign treatment priorities: the Australasian Triage Scale (ATS), the Canadian Triage and Acuity Scale (CTAS), the Manchester Triage System (MTS), and the Emergency Severity Index (ESI) [5-16]. There is, indeed, some data from secondary health care systems suggesting that team-triage may reduce waiting times to see a doctor and to radiology and the length of stay in the ED [17]: experienced doctor-nurse triage teams have been reported to be an effective way of shortening the waiting time in the ED, irrespective of the urgency of the condition.

The health system in Finland is divided into private and public primary care (GP) services and in addition to primary care ED and secondary care ED services. EDs and most of the office-hours primary care are funded by the public health system. In other words, they are non-profit making. Emergency services in Finland have been provided by both hospitals and health centres since the 1970s. Out-of-hours services in health centres are run by primary health care staff and GPs while the EDs of the secondary care hospitals are run by different medical specialities. Primary care out-of-hours units were increasingly incorporated into hospital emergency units due to centralization at the end of the 20th century. These EDs came to be known as 'combined emergency departments' [16]. GPs are responsible for the initial assessment and treatment in the EDs, thereby regulating access to the acute secondary health care. One argument for this centralization is that a considerable number of patients needing acute care, also require hospital treatment, tests performed in hospital and medical attention from specialists [16]. The use of out-of-hours services decreased when the service of the public primary health care centres was improved in the 1990's by the so-called personal doctor system [18]. Decreased use of EDs indicated that a smoothly running public service during office hours reduced the demand for out-of-hours services [18]. This is observed to be a general trend when the quality of daytime primary care is adequate [19]. As a complementary, profit driven system there is a well-equipped private primary health care which is, however, more expensive for the patients to use. Patients choosing this system cover the expenses by using their own money and insurances. Both the public and private sector primary care and private secondary care consult public secondary care by using referrals. The most difficult clinical cases are usually treated in public secondary care.

The situation in Finnish primary care has recently deteriorated due to difficulties in recruiting GP:s into the public health system. As a consequence, access to public daytime services has worsened [18] and EDs are forced to back up the inadequate daytime services in primary and secondary care. Easily accessible EDs may also be considered as an extra public service for those who are, for various reasons [4], not willing or able to use daytime services. The EDs are overused and this situation has led to negative patient feedback and increased frustration among the staff [20]. There have been difficulties in recruiting doctors and a growing tendency to outsource the work of the GPs to agency employees. This is partly due to the nature of the work and inconvenient working hours, [18,20]. Therefore, the turnover of primary care doctors especially in out-of-hours services has been high [18]. It has also been difficult to recruit experienced nursing staff to the emergency system. Many stakeholders and organizations are involved in the provision of emergency services making the responsibility for the leadership and the development of the EDs unclear.

Emergency services must be capable of providing quick, high quality and effective treatment to patients with acute medical problems. This capability is compromised if the ED is too crowded [21]. Internationally, most countries separate primary care and ED services and define ED services as secondary care functions and EDs have their own triage scales [5-15]. In Finland, there are also primary care EDs and this is the main reason for developing a specific triage scale for primary health care ED's. As an attempt to provide immediate treatment for those patients in primary health care EDs who need it the most, a face-to-face triage system [16] based on letters from A, B, C, D and E for assessing the urgency of patients' treatment needs was applied in the main combined ED in the City of Vantaa, Finland (Peijas Hospital). In this system, all patients who were transported to the ED by ambulance were triaged by secondary health care nurses consulting secondary doctors for safety reasons. Patients arriving by other means than ambulance go to primary health care ABCDE-triage. Patients in group E are not in need of urgent medical treatment. At least 8% of primary care ED patients have been reported to belong to this group [16]. Yet it is very important to be sure that also patients in this group are in safe hands and can trust that their evaluation in ED is made by approved standards. In most cases they are treated by nurses.

The primary aim of the present study was to determine whether this type of triage system combined with public guidance related to the proper use of EDs alters the patient flow (monthly number of visits) in the GP driven department (primary health care) and the specialist driven (secondary health care) ED also in other systems and in other municipalities than the city of Vantaa. A secondary aim was to study if the introduction of ABCDE-triage in the ED alters the number of patient visits in other public or private primary health care services. To know what kind of cases there are in E group, we also recorded the patient's reasons for entry to the ED.

## Methods

### Sample

This study was performed in the city of Espoo. At the time of the study Espoo had a population of around 230,000 inhabitants. In the present work, unselected primary care patients constituted the study population. Intervention was performed and data collected from the primary care led ED:s. One of them is located in Jorvi hospital. Since secondary health care is also provided in Jorvi it is defined as a combined ED. It is equipped with out-of-hours laboratory and X-ray facilities, and primary care ED is carried out there only out of office hours. The other ED in Puolarmetsä is more like a traditional Finnish primary health care out-of-hours unit. There is no specialist care provided, and the laboratory and X-ray facilities are available only during office hours. Puolarmetsä ED was not open during the night-time but only in the evenings and at weekends. Altogether, the data obtained from Jorvi and Puolarmetsä EDs were pooled together as Espoo ED's data to study the effect on the patient currents in different main compartments of the local health care.

### Variables

The data was obtained from the electronic health records of Espoo primary health care (Effica- patient chart system) and Jorvi secondary health care ED (Helsinki University Central Hospital, HUCH; Oberon- patient chart system). The Social Insurance Institution of Finland (SII) provided the data about the use of the private primary health care doctors. In Espoo, the follow-up was performed between March 2004 and February 2008. The number of monthly visits to doctors was scored in each study department before and after implementation of the ABCDE triage system (1.3. 2007). Thus, we could study the situation before and after the implementation of ABCDE-triage in the EDs. In the case of those patients allocated to triage group E, the reasons for entry to the primary care EDs were recorded by using ICPC 2 (Finnish ICPC 2, 2010, http://www.kuntaliitto.fi) classification that was performed by the triage nurses. No ethical approval was required because this study was made directly from the patient registry without identifying the patients. The registry keeper (health authorities Espoo and HUCH) granted permission to do the study.

### Intervention

The intervention was part of a larger project aimed at improving the quality of ED services and reducing waiting times [16]. The leaders of the project analyzed the process. ABCDE-triage [16] was performed by experienced nurses in the frontline. Almost 60 nurses were educated by the medical directors (RM and JK) of the project to perform the ABCDE triage. These nurses assessed the patients before attending the doctor. The patients were triaged subjectively by the nurse as shown in Table 1. During the first seven months, the non-urgent (group E) patients were given the option of waiting to be seen by the doctor, but without any promises about how long the waiting time would be. Later on, they were redirected home with self-care advice and advice to contact day-time services if the symptoms persisted. If the status of the patient altered in the waiting room a re-triage was performed. If a nurse was uncertain about her assessment she could ask for advice from a doctor or assess the patient in the higher triage group. Those patient groups who would need special attention were identified based on interviews with different specialists and stakeholders. These groups were identified as the following: elderly people, children and people suffering from mental illness or drug abuse.

**Table 1:** The 5 (five) scale groups from A to E used at Peijas and Jorvi ED

A	immediate care (for example resuscitation)
B	the patient must be seen by a doctor (usually a specialist) within 10 minutes (acute crises)
C	the patient needs to meet a doctor within 1 hour (severe infections, trauma etc)
D	the patient needs to meet a doctor within 2 hours (minor trauma, less severe infections etc)
E	Not an acute patient, redirect or must wait until more urgent patients from groups A-D were treated (non-urgent problems: mild upper respiratory tract infections, mild fever, cough, chronic symptoms in back, pain in ear, mild diarrhea or vomiting, prolonged general weakness and tiredness)

The impact of introducing the ABCDE-triage tool in emergency services was enhanced by increasing simultaneously the education of the staff in EDs and the publicity about the issue. A discussion was also raised in the media around these services and information was delivered both to professionals and the public outside EDs. The main message to the public was that those who require immediate medical help should come to EDs but EDs are not overflow services of office-hour services. Guidelines were written for the staff about triage. The staff also got training and encouragement by the project workers and leaders. The training was arranged for public health care inside EDs and in office-hour services. Altogether 60 nurses were trained in four 4-hour seminars in primary care EDs to perform the triage. The general public was informed of the project through the media, and all the information focused on the transparency of the system. Necessary data was also available via Internet, and both the public and staff had access to the internet pages of the campaign http://www.hus.fi/default.asp?path=1,32,660,546,570,4384,6950,6956,11437. All related material was, and is still, available at this page. Local print media, radio and bulletins were also used. About thirty articles were published in both national and local newspapers. Posters and leaflets about the project were delivered to the patients in EDs and in office-hour services. The aim of the project group was to publish as much information as possible related to the changes to keep the population, all organizations associated with the project and the staff fully informed. The objective of this information campaign was to guide non-acute patients (group E) directly to appropriate daytime services. There was also lively public and political debate about emergency services going on at the same time. Feedback was actively gathered both from patients and the staff with questionnaires and interviews. The Numbers of visits to doctors and nurses and assessed patients were frequently measured. Similarly, patients assessed in triage groups, waiting times and diagnoses in different triage groups were irregularly measured. In order to discuss the implementation process and problematic patient cases, follow-up meetings were organized every month.

### Statistical analysis

The triage system was introduced at the beginning of March 2007. The frequencies of monthly patient visits in the three previous years were compared to the number of patient visits in the respective months of the next year (March 2007-February 2008), e.g. after the triage was applied. One-way ANOVA of repeated measurements followed by t-test with the Bonferroni Correction was chosen as the method for statistical analysis. [16]. After implementation of the triage, direct and proportional distributions of the reasons for entry to the ED were recorded by using ICPC 2 classification in group E patients for ten months.

## Results

**Figure 1:**
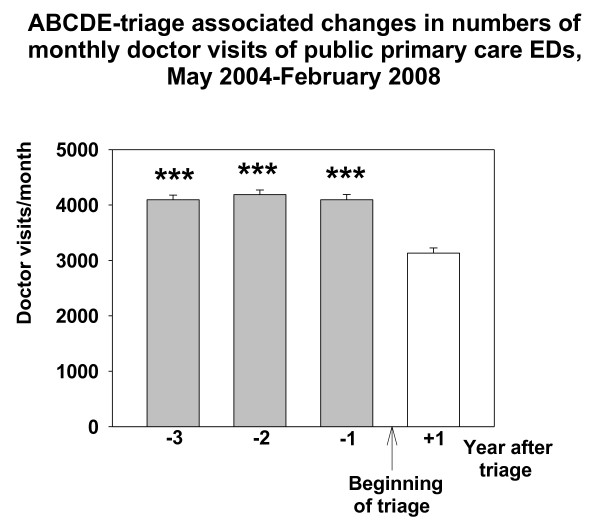
**ABCDE -associated changes in numbers of monthly doctor visits in EDs of Espoo**. Data are shown as one year epochs before and after triage. Mean and SE (brackets) is shown. *** means P < 0.001, Bonferroni test compared with the frequency of monthly visits in the one year epoch after beginning the ABCDE-triage.

**Figure 2:**
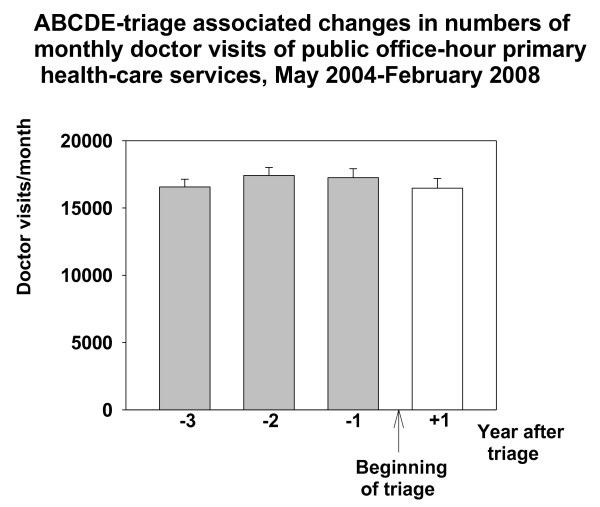
**ABCDE -associated changes in numbers of monthly office-hour doctor visits in Espoo**. Data are shown as one year epochs before and after triage. Mean and SE (brackets) is shown.

**Figure 3:**
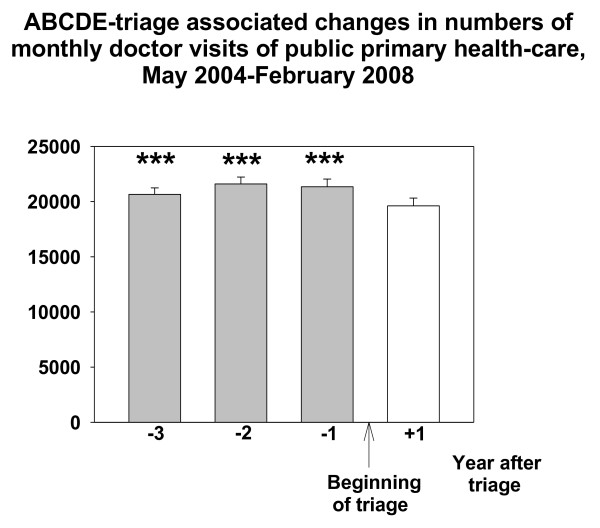
**ABCDE -associated changes in numbers of monthly doctor visits in public side of GPs in Espoo**. Data are shown as one year epochs before and after triage. Mean and SE (brackets) is shown. *** means P < 0.001, Bonferroni test compared with the frequency of monthly visits in the one year epoch after beginning the ABCDE-triage.

Three years (May 2004-February 2005) before intervention the number of doctor visits in Espoo Primary care EDs was 49141, two years (March 2005-February 2006) before 50248, and one year (March 2006-February 2007) before 49219, respectively. The number of these visits one year (March 2007-February 2008) after beginning of the triage (1^st ^March 2007) was 37589. The number of the monthly GP doctor visits in the ED (out-of-hours) decreased by about 24% (962 visits/month) from the numbers of the last control year (March 2006-February 2007) after the introduction of the ABCDE- triage system (RM-ANOVA, F{11,3} = 77.191, p < 0.001, Figure 1). At the time of the introduction of triage in Espoo EDs, there was no change in the number of monthly doctor visits in office-hour public services (mean; 16565-17414 visits/month, Figure 2). The Total number of monthly doctor (GP) visits in the whole public health care system decreased after the implementation of the ABCDE-triage by 8.1% (RM-ANOVA, F{11,3} = 29.145, p < 0.001, Figure 3)

**Figure 4:**
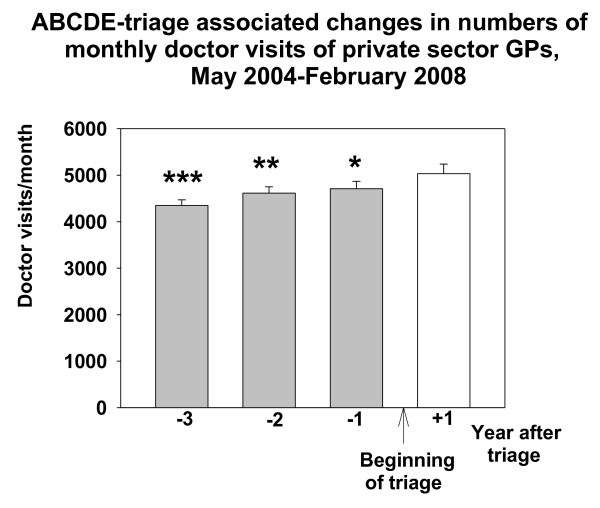
**ABCDE-associated changes in numbers of monthly doctor visits in private sector GPs in Espoo**. Data are shown as one year epochs before and after triage. Mean and SE (brackets) is shown. * means P < 0.05, ** P < 0.01 and *** P < 0.001, Bonferroni test compared with the frequency of monthly visits in the one year epoch after beginning the ABCDE-triage.

**Figure 5:**
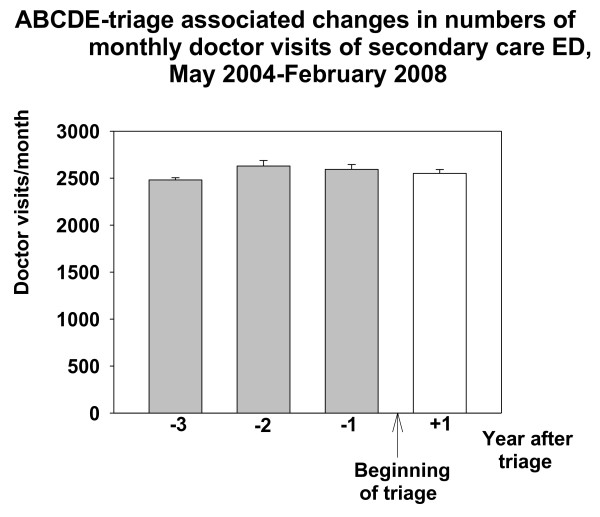
**ABCDE-associated changes in numbers of monthly visits and referrals to secondary health care in Jorvi ED**. Data are shown as one year epochs before and after triage. Mean and SE (brackets) is shown.

Doctor visits to the private sector GP:s in Espoo increased after the beginning of the intervention. This increase was about 324 visits/month when compared with the number of doctor visits of the last control year (March 2006-February 2007) before implementing the triage (RM-ANOVA F{11,3} = 14.387, p < 0.001, Figure 4). The number of doctor visits in secondary health care ED in Jorvi hospital (HUCH) did not change after the implementation of triage in primary health care EDs (Figure 5).

Reasons for entry to the Espoo EDs recorded by using ICPC 2 classification are shown in Table 2. The Most common reasons allocated to group E in both ED's were respiratory (28,5%), musculoskeletal (15,6%), general and unspecified (13,7%) and skin symptoms (10,5%).

**Table 2:** Distribution of the reasons to entry the ED recorded by using ICPC 2 classification in group E patients (N = 1244) of the Espoo EDs

ICPC Classification		%	N	ICPC Classification		%	N
**R**	**RESPIRATORY**	**28,50%**	**354**	**D**	**DIGESTIVE**	**7,50%**	**93**

R05	Cough		61	D01	Abdominal pain/cramps general		14
R21	Throat symptom/Complaint		75	D10	Vomiting		13
R29	Respiratory symtom/Complaint other		9	D11	Diarrhoea		25
R74	Upper respiratory infection acute		146	**H**	**EAR**	**5,60%**	**70**
R75	Sinusitis acute/chronic		11	H01	Ear pain/Earache		36
R97	Allergic rhinitis		10	H27	Fear on ear disease		8
**L**	**MUSCULOSKELETAL**	**15,60%**	**194**	**F**	**EYE**	**4,80%**	**60**
L01	Neck symptom/Complaint		134	F02	Red eye		14
L02	Back symptom/Complaint		64	F29	Eye symptom/Complaint other		20
L05	Flank/Axilla symptom/Complaint		6	F73	Eye infection/Inflammation other		8
L08	Shoulder symptom/Complaint		6	**-**	**PROCESS CODES**	**3,50%**	**44**
L09	Arm symptom/Complaint		6	**P**	**PSYCHOLOGICAL**	**3,30%**	**41**
L14	Leg/Thigh symptom/Complaint		7	P15	Chronic alcohol abuse		10
L15	Knee symptom/Complaint		12	P16	Acute alcohol abuse		6
L16	Ankle symptom/Complaint		6	**N**	**NEUROLOGICAL**	**2,30%**	**29**
L17	Foot/Toe symptom/Complaint		19	**U**	**UROLOGICAL**	**2,10%**	**26**
**A**	**GENERAL AND UNSPECIFIED**	**13,70%**	**170**	**X**	**FEMALE GENITAL**	**1,10%**	**14**
A02	Chills		7	**K**	**CARDIOVASCULAR**	**0,80%**	**10**
A03	Fever		105	**W**	**PREGNANCY, CHILDBEARING, FAMILY PLANNING**	**0,40%**	**5**
A29	General symptom/Complaint, other		9	**Z**	**SOCIAL PROBLEMS**	**0,20%**	**3**
A80	Trauma/Injury NOS		7	**Y**	**MALE GENITAL**	**0,10%**	**1**
**S**	**SKIN**	**10,50%**	**130**				
S08	Skin colour change		8				
S09	Infected finger/toe		8				
S10	Boil/carbuncle		6				
S11	Skin infection post-traumatic		6				
S16	Bruise/contusion		10				
S18	Laceration/cut		14				
S19	Skin injury other		7				
S29	Skin symptom/complaint other		9				

Total and proportional number of visits including in different main ICPC-groups (A-W) are given. Respectively, the most common ICPC-coded reasons (> five visits) for entry to the EDs during the follow up period (March-December 2007) are separately presented.

## Discussion

The implementation of the ABCDE-triage combined with public guidance was associated with reduction in the number of patient visits to GP out-of-hours ED services by about 24%. The observed reduction in GP visits in the ED may partly be due to considerable public debate and the publicity provided by the new system and rules. It is possible that some of the patients decided not to request emergency care at all due to the expected long waiting times or risk of being redirected to daytime health services. Patients were also assessed to group E by the triage nurse and redirected to homecare. This result is higher than our former experience from Vantaa City where the number of ED visits decreased by 8% after implementation of ABCDE-triage [16]. In Espoo, the population seemed to adapt very quickly to the idea that those who needed help most must go first and those whose need is not urgent should not necessarily visit the ED at all. However, a considerable difference between Vantaa and Espoo was, that in Espoo the patient who was assessed to group E might be sent home with advice while in Vantaa the patient was allowed to stay and wait as long as the queue of more urgent patients (groups A-D) persisted [16]. This may also have explained why the decrease in patient visits was much higher in Espoo than in Vantaa.

GPs were previously assumed to regulate access to the acute secondary health care by referring those patients who need specialist care. The triage was performed by primary health care in EDs but it did not diminish or increase the workload of the secondary health care in the same facility. Altogether, the present finding agrees with the former report of Vertesi [3] which suggested that triage did not automatically enhance activities in the secondary health care ED.

The number of visits to primary care GPs during office hours was unchanged from March 2004 to February 2008 in Espoo (Figure 3). Thus, the decrease in patient visits to the GPs in Espoo EDs did not cause an overflow of patients in the office hour GP practice. There were some hints that demand for nurse visits in daytime services increased but this could not be verified because also other changes were made in office-hour public health to alter the workload of nurses at the same time. Furthermore, no excessive doctor resources were allocated to office-hour activities at the time of the intervention. Thus, we cannot exclude the possibility that the lack of change in the number of visits to primary care GPs during office hours was just attributable to that fact. Yet the same phenomenon was observed in Vantaa City in our previous work [16]. Thereby, our results are in line with the suggestion that EDs also have "customers of their own" and that those patients are not likely to use ordinary daytime primary health care services [4].

Even the total number of visits to GPs in the public health system was reduced after implementation of the ABCDE-triage combined with public guidance in Espoo EDs. As a probable compensation for this decrease, the number of visits to the private sector GPs increased after the triage was applied in Espoo (Figure 4). There has been reported to be a correlation between public and private sectors with respect to the demand for health care and health care utilization [22]. If the supply of public health care is considered to be unsatisfactory patients look for care in the private sector [23]. Such a shift may have been observed in the current study, too. When access to EDs was limited for non-urgent patients, part of them probably sought help from the private sector. This was different from our former observation in Vantaa where no such shift to the private sector was seen [16]. Speculatively, the explanation for this difference could be the fact that people in Espoo are more used to visiting the private sector than in Vantaa [16]. Furthermore, the inhabitants in Espoo are somewhat wealthier than those of Vantaa [24] and therefore more able to use relatively expensive private primary care. Of course, other possible confounding factors may exist. Changes in the economic situation and occupational health care, supply of services in private health care and occupational health care might alter the use of primary health care. However, there are no published data to support the impact of these latter factors.

Yet, we cannot rule out that very strict ABCDE-triage could result in inequality in obtaining health services in society. In Peijas ED, use of ABCDE-triage without the possibility of sending E group away from the ED without seeing the doctor did not increase the use of primary health care [16]. On the other hand, this action was associated with a reduction in visits to the doctor by only 8% [16]. In Espoo EDs, most redirected patients seemed to have relatively self-limiting harmless conditions as can be seen from the ICPC-2 classification of the entry reasons in group E (Table 2). Guidelines have been revised from this perspective and the information flow from ED to daytime services (both medical and social) has been enhanced and made systematic. Further studies will have to be carried out to study how well the present system supports these special groups.

In patients allocated to group E, the most common reasons for entry to the EDs were respiratory, musculoskeletal, general and unspecified and skin symptoms. Patients complaining of neurological and cardiovascular symptoms were rarely allocated to E group indicating relative reliability of ABCDE-triage. Thus, the triage protocol was well followed by the trained nurses. Furthermore, nurses should be able to evaluate the severity of these clinical states. However, patient safety issues are essential when applying triage in an ED. The key player in our triage model is the nurse who makes the assessment of the patient upon arrival. In many EDs around the world triage has been successfully run by experienced nurses [25,26]. Furthermore, there are reports suggesting that some activities formerly performed by physicians in primary health care were safely performed by trained nurses [27]. Interestingly, no short term excessive mortality or excessive amount of adverse events was observed among patients who left the ED without being examined by a doctor in a Canadian study [28]. Indirectly, this suggests that if a patient of an ED triages himself to a group resembling our E-group (leaves without seeing by a doctor) his risk of having a severe acute health problem is low.

Emergency departments around the world use different triage systems to assess the severity of incoming patients' conditions and assign treatment priorities: the Australasian Triage Scale (ATS), the Canadian Triage and Acuity Scale (CTAS), the Manchester Triage System (MTS), and the Emergency Severity Index (ESI) [5-14]. Triage instruments with 5 levels have been suggested to be superior to those with 3 levels in both validity and reliability and good to very good reliability has been shown for the best-studied instruments, CTAS and ESI, while ATS and MTS have been found to be only moderately reliable [15]. In Finland, the most used triage system in is the five-level ABCDE triage instrument [16 and Table 1]. It is developed for the use of primary health care ED's and differs from hospital oriented triage systems (ATS, CTAS, MTS and ESI) (Table 3). In ABCDE-triage A-group patients go straight into secondary care and BCDE-patients into primary care ED. Nurses take care of E-group patients. There is neither ESI resource nor MTS stream-line thinking. Only MTS contains components where patients are redirected to primary care by supporting a "Presentation-Priority Matrix". In this matrix, there are 50 presentations with 5 priorities = 250 or so "boxes" which can be mapped by consensus onto particular "streams". The triage nurse will place patients in the identified area of the service [13]. Streams are mainly in secondary care. For those patients triaged to primary care the triage nurse identifies this group of patients but even in this system it will be clinicians in the Urgent Care Centre (UCC), ENPs and APs, who carry out the actual deflection. In ABCDE-triage the waiting time is the most important factor in streaming the patients.

**Table 3:** Comparison of 5 Level Triage scales [ ABCDE, the Emergency Severity Index (ESI),), the Manchester Triage System (MTS), the Canadian Triage and Acuity Scale (CTAS) and the Australasian Triage Scale (ATS) ]

	Primary Health	Hospital ED	Validity and Reliabity	Vital signs	Acuity-based	Resource- based
	Care ED		Research			
ABCDE	X	-	-	-	X	-
ESI	-	X	X	X	X	X
MTS	-	X	X	X	X	-
CTAS	-	X	X	X	X	-
ATS	-	X	X	X	X	-

The idea in the ABCDE-triage system is to evaluate and treat those primary health care patients who usually are at low risk and who come to ED's with a reasonable use of resources. Stronger scientific evidence is needed to determine which of the vital signs and chief complaints have the greatest prognostic value in the triage. Patients may have a life-threatening condition, but show normal vital signs. Inter rater agreement (reliability), validity, and safety of triage scales need to be investigated further, and head-to-head comparisons are needed to determine whether any of the triage scales have advantages over others [29]. Nevertheless, the quality of triage assessment must be continuously monitored and the number of incorrect assessments minimized. Right now further studies are ongoing on the safety of the present ABCDE-triage system and also on the changes in waiting time associated with triage. It would also be interesting to know more about the patient flows, such as the destination of the patients, and whether these flows changed after implementation of the ABCDE triage. With destination is here meant patient flows to secondary ED, hospital admissions, patients treated at the primary ED and then sent home, or sent home immediately (= urgency group E). This lack of information is a considerable limitation of our study. We are planning to perform studies aimed to reveal above mentioned patient flows more profoundly in the future.

## Conclusion

Implementation of ABCDE-triage combined with public guidance was associated with a reduction in the use of a primary health care ED services. This intervention did not seem to increase the workload during office hours in the public primary health care or in secondary health care. Strict ABCDE-triage combined with public guidance may decrease total use of public primary health care and redirect part of the patients to the private sector.

## List of abbreviations

ED: Emergency department; GP: General practitioner.

## Competing interests

The authors declare that they have no competing interests.

## Authors' contributions

JaK led and performed the intervention planned the analysis and wrote the manuscript. RM led and performed the intervention and arranged the data from Espoo. TH led and performed the intervention. JM arranged the data from tertiary health care. KJM planned the analysis and wrote the manuscript. TK arranged the data from the private sector, analyzed the data, planned the experimental setting and wrote the manuscript. All the authors have read and approved the final manuscript.

## Pre-publication history

The pre-publication history for this paper can be accessed here:

http://www.biomedcentral.com/1471-227X/12/2/prepub
